# Inequalities in mental health, self-rated health, and social support among sexual minority young adults during the COVID-19 pandemic: analyses from the UK Millennium Cohort Study

**DOI:** 10.1007/s00127-022-02291-1

**Published:** 2022-05-04

**Authors:** Laia Bécares, Dylan Kneale

**Affiliations:** 1 Department of Social Work and Social Care, University of Sussex, Essex House, Falmer, Brighton, BN1 9QQ UK; 2 EPPI-Centre, UCL Social Research Institute, University College London, London, UK

**Keywords:** Sexual minority, Mental health, Coronavirus pandemic, Social support, Self-rated health

## Abstract

**Purpose:**

Young adults who self-identify as a sexual minority may have been particularly harmed by the consequences of lockdown, closure of educational institutions, and social distancing measures as they are likely to have been confined in households that may not be supportive of their sexual orientation. We examine inequalities in the mental health and self-rated health of sexual minority young adults, compared to their heterosexual peers, at the height of lockdown restrictions in the UK.

**Methods:**

We analysed data from singletons who participated in waves 6, 7, and the wave 1 COVID-19 survey (*n* = 2211) of the Millennium Cohort Study, a nationally representative longitudinal study of infants born in the UK between September 2000 and January 2002. Regression models compared the mental health, self-rated health, and social support of sexual minority young adults to that of their heterosexual peers.

**Results:**

One in four young adults self-identified with a sexual orientation or attraction other than completely heterosexual. Sexual minority young adults had significantly lower levels of social support (*β* =  − 0.38, SE 0.08), poorer self-rated health (OR 3.91, 95% CI 2.41–6.34), and higher levels of psychological distress (*β* = 2.26, SE 0.34), anxiety (*β* = 0.40, SE 0.15), and loneliness (*β* = 0.66, SE 0.18) when compared to heterosexual young adults.

**Conclusions:**

Sexual minority young adults in the UK have been detrimentally impacted by the coronavirus pandemic, experiencing inequalities in mental health, self-rated health, and social support when compared to heterosexual young adults. Implications for policy and practice include a stronger provision of safe spaces in the community and in institutions, and policies that address marginalisation and harassment.

## Introduction

The COVID-19 pandemic has magnified existent social inequalities, detrimentally impacting communities that were already disadvantaged by enduring institutional and systemic oppression. This has been acutely felt by people who self-identify as Lesbian, Gay, Bisexual, and Queer, and who identify in another way other than heterosexual (hereby called sexual minority) [1]. Although data on COVID-19 infection and mortality among sexual minority people are not widely available due to lack of measurement in electronic health records or public health surveillance, available global evidence indicates that sexual minority populations have been disproportionately impacted by the COVID-19 pandemic compared to their heterosexual peers in relation to mental health [2–5], health care access [6], and social and economic outcomes [7].

Sexual minority young adults may have been particularly harmed by the consequences of lockdown, closure of educational institutions, and social distancing measures as they may have been confined in households that may not be supportive of their sexual orientation. They have also experienced disruption in a significant life course stage of identity formation where sexual minority young adults may have been preparing to or dealing with ‘coming out’ to family and friends. Evidence from international studies has provided initial support for this, but findings come from small samples using convenience sampling or qualitative methods [8–10], or do not specifically focus on young adults [2]. For example, Fish and colleagues describe the intrapersonal, interpersonal and structural challenges that young sexual minority adults faced in their study, with participants discussing how the confines of lockdown prevented accessing appropriate support, and the enforced concealment of identity [9]. Despite the richness of their findings, the study design and methodology does not allow for the magnitude of inequalities between sexual minorities in terms of physical and mental health to be estimated.

In this study, we analyse data from a UK nationally representative dataset to examine inequalities in the impact of the COVID-19 pandemic on the health and mental health among young adults who self-identify with a sexual orientation other than heterosexual/straight, compared to their heterosexual/straight peers. We also assess the role of social support and economic adversity and other sociodemographic factors in explaining possible unequal outcomes among sexual minority young adults.

The purpose of this study is not to estimate the causal impact of the COVID-19 pandemic in exacerbating pre-existing inequalities (or less likely in attenuating inequalities); rather the focus here is to estimate the magnitude of inequalities during the first wave of the pandemic in the UK (May 2020). Understanding the scale of the gulf between the mental health of young sexual minority people and that of heterosexual young people is of key social policy relevance as governments in the UK and elsewhere consider how to strengthen mental health supports and systems. Robust evidence from nationally representative data demonstrating the extent of the magnitude of sexuality-based inequalities during the height of the pandemic can inform on where and how some of this support should be directed. Our focus on social support as a key moderator of inequality is aligned with policy interest around interventions that are scalable and adaptable to the pandemic to improve population-level mental health [11].

## Methods

### Study design and participants

The Millennium Cohort Study (MCS) is a nationally representative longitudinal study of infants born in the UK between September 2000 and January 2002. Families with children who were living in the UK at 9 months were identified through the Department of Work and Pensions Child Benefit system (a universal benefit in the UK) and selected on the basis of where the family was resident shortly after the time of birth. The sample is stratified by ethnicity and disadvantage, and clustered at the electoral ward such that disadvantaged residential areas and areas with a high proportion of ethnic minority people are over-represented. More detail on the survey design, recruitment process and fieldwork can be found elsewhere [12].

There have been data collection sweeps when cohort members were aged about 9 months (MCS1), 3 years (MCS2), 5 years (MCS3), 7 years (MCS4), 11 years (MCS5), 14 years (MCS6), and 17 years (MCS7). Since the start of the coronavirus pandemic, there have been three waves of additional data collection (COVID-19 surveys). The first wave of the COVID-19 survey took place in May 2020, at the height of the first lockdown restrictions in the UK. The survey focused mainly on how participants’ lives had changed from just before the outbreak of the pandemic in March 2020 up until their response to the survey. The response rate of the wave 1 COVID-19 survey is 26.6% (*N* = 2645). In the present study, we use data from singletons who were productive in waves MCS6, MCS7, and the wave 1 COVID-19 survey (*n* = 2211). Outcome variables were, therefore, assessed when the majority of participants were aged 19 years.

### Variables

Sexual orientation was measured by combining two variables asked in MCS7 that captured participants’ sexual orientation and sexual attraction. Participants were first asked how they currently thought of themselves, with five response categories ranging from ‘completely heterosexual/straight’ to ‘completely gay or lesbian.’ Respondents were also asked who they felt sexually attracted to, with six response categories ranging from ‘only to opposite sex, never to same sex,’ to ‘only ever to same sex, never to opposite sex.’ Our measure of sexual orientation combines sexual orientation and attraction into a binary variable that classifies participants who selected both a completely straight sexual orientation and a sexual attraction only to the opposite sex into the reference category of ‘straight/heterosexual.’ Participants who self-identified with a sexual orientation other than completely heterosexual/straight, and who reported a sexual attraction other than solely to the opposite sex were classified as a sexual minority.

Given the relatively small sample size of the MCS COVID-19 study, we were not able to disaggregate individual groups (for example, bisexual, gay/lesbian). Likewise, although the MCS asks information that would allow us to examine inequalities among trans and gender diverse young people compared to their cisgender counterparts, we do not have sufficiently large sample sizes to appropriately consider sexual orientation and gender identity in our analyses, so the present paper focuses on sexual orientation only.

All measures of health, mental health, and loneliness come from the wave 1 COVID-19 survey.

Health was measured using overall self-rated health, which has been shown to be a valid indicator of health status. Reports of poor health have been associated with higher mortality, psychological distress, and poor functioning [13, 14]. In the MCS, respondents were asked to rate their health on a scale ranging from 1 (excellent) to 5 (poor). We dichotomised responses into excellent, very good, and good, or fair and poor health.

We used the Kessler 6 (K6) scale to identify psychological distress [15]. This measure asks how often respondents felt nervous, depressed, hopeless, restless or fidgety, worthless, or that everything was an effort in the last month. Respondents answered on a five-point scale from 1 (all the time) to 5 (none of the time). We reversed and rescaled all items from 0 to 4 so that high scores indicate high levels of psychological distress. Total scores for this measure can range from 0 to 24, with higher scores indicating greater psychological distress. The K6 scale has good internal consistency (Cronbach’s *α* = 0·87) in the study sample. We modelled the K6 scale as a continuous measure, and also report the descriptive prevalence of severe psychological distress using a binary variable where we dichotomised the K6 at the recommended cut-off score of 13 [15].

We measured anxiety symptoms using the two-item Generalized Anxiety Disorder (GAD2) questionnaire [16], which assesses the presence and severity of anxiety symptoms within the past 2 weeks. GAD2 includes two items asking respondents whether they have been bothered by problems—feeling nervous, anxious or edge, or not being able to stop or control worrying—over the last 2 weeks, each scored from 0 (not at all) to 3 (nearly every day). We summed the two variables to compute a total score, with higher values indicating worse anxiety symptoms (Cronbach’s *α* = 0·86). We modelled the GAD2 as a continuous measure in the main analyses, and also report the descriptive prevalence of clinically significant anxiety symptoms using a binary variable where we dichotomised the GAD2 at the recommended cut-off score of 3 [16].

To measure loneliness we used the three-item UCLA Loneliness Scale [17], and an additional item that asked respondents how often they feel lonely (responses including hardly ever, some of the time, often) which has been used in other UK cohort studies. The three items from the UCLA Loneliness scale assessed the frequency in response to questions about feeling lack of companionship, feeling left out, and feeling isolated from others. Response categories were the same for all four items measuring loneliness. We summed the four items to create an overall loneliness score, with higher values capturing increased loneliness (Cronbach’s *α* = 0·83).

Availability of social support was measured with the three-item Short Social Provisions Scale [18], which asks respondents to think about their current relationships with friends, family members, community members and so on. Respondents were asked to indicate the extent to which they have family and friends who help them feel safe, secure and happy; there is someone they could turn to for advice if they were having problems; and there is no one they feel close to (reverse ordered). Response categories ranged from very true to not true at all. We summed the individual items to create a measure of availability of social support, with higher values indicating higher social support (Cronbach’s *α* = 0·69).

We adjusted for variables thought to confound the association between our exposure and outcome variables. This included age, gender, region of residence, ethnicity, and equivalised household income. Cohort member’s ethnic group and equivalised household income were taken from data collected at MCS6.

#### Statistical analysis

We examined outcomes and explanatory factors by sexual orientation using bivariate analyses to provide descriptive statistics on the social support, health, mental health, and socioeconomic characteristics of the MCS sample. We used regression analyses to explore the association between sexual orientation and the five outcomes (social support, self-rated health, psychological distress, anxiety, and loneliness). We constructed binary logistic regression models for self-rated health, and Ordinary Least Squares regression models for continuous measures of social support and mental health. We built model sequentially to explore the contribution of different explanatory factors to inequalities in sexual orientation on the impact of the coronavirus pandemic. The baseline model (Model 1) examined the unadjusted association between sexual orientation and the different outcomes. Model 2 examined health inequalities across sexual orientation adjusted for gender and age. Model 3 further adjusted for ethnicity, region of residence, and equivalised household income. In models that examined the association between sexual orientation and health and mental health, we had a further model (Model 4) that additionally adjusted for social support. All models used heterosexual young adults as the reference category. We analysed data using the ‘svy’ commands in Stata version 16 [19]. All analyses were based on complete cases and were weighted to take account of the stratified and clustered sample design, and the unequal probability of being sampled.

## Results

One in four young adults self-identified with a sexual orientation or attraction other than completely heterosexual/straight. There were a higher proportion of women in the sexual minority group, compared to the heterosexual group (57% vs 46%). A lower proportion of young adults who self-identified as a sexual minority also self-identified as an ethnic minority, compared to young adults who self-identified at heterosexual/straight (see Table 1). In unadjusted models, sexual minority young adults had lower levels of social support, poorer self-rated health, and poorer mental health than their heterosexual peers (see Table 1).

**Table 1:** Sample characteristics of the Millennium Cohort Study

	Heterosexual	Sexual minority	Total
Weighted	(*n* = 1369)	(*n* = 463)	(*N* = 1832)
Unweighted	(*n* = 1415)	(*n* = 547)	(*N* = 1962)
Gender			
Men	743 (54)	199 (43)	942 (51)
Women	626 (46)	264 (57)	891 (49)
Ethnic group			
White	1154 (84)	413 (89)	1567 (86)
Mixed	59 (4.4)	24 (5.3)	84 (4.5)
Indian	–	–	26 (1.3)
Pakistani or Bangladeshi	–	–	57 (3)
Black	–	–	37 (2)
Other	45 (3.4)	16 (3.5)	61 (3.2)
Region			
London	141 (10)	47 (10)	189 (10)
North East	28 (2)	14 (3)	41 (2.3)
North West	123 (9)	47 (10)	170 (9.3)
Yorkshire and the Humber	117 (8.5)	41 (8.8)	157 (8.6)
East Midlands	131 (9.6)	28 (6)	159 (8.7)
West Midlands	89 (6.5)	34 (7.4)	124 (6.7)
East of England	158 (12)	50 (11)	208 (11)
South East	261 (19)	85 (18)	346 (19)
South West	136 (10)	46 (10)	183 (10)
Wales	60 (4.4)	20 (4.3)	81 (4.4)
Scotland	87 (6.4)	37 (8.1)	125 (6.8)
Northern Ireland	37 (2.7)	14 (3)	51 (2.8)
Equivalised household income			
Lowest quintile	128 (10)	67 (14)	195 (11)
Second quintile	178 (13)	60 (13)	238 (13)
Third quintile	243 (18)	75 (16)	318 (17)
Fourth quintile	376 (27)	107 (23)	483 (26)
Highest quintile	445 (33)	154 (34)	599 (33)
Social support, M (SD)	5.46 (0.98)	5.10 (1.28)	5.35 (1.09)
Self-rated health			
Excellent, very good, or good	1290 (94)	370 (80)	1660 (91)
Fair or poor	79 (6)	93 (20)	173 (9)
Psychological distress, M (SD)	6.92 (2.35)	8.02 (0.20)	7.20 (0.08)
Anxiety, M (SD)	7.04 (4.60)	10.22 (5.54)	7.88 (5.01)
Loneliness, M (SD)	1.55 (1.68)	2.20 (1.90)	1.71 (1.75)

Data are *n* (%), unless otherwise indicated. Some estimates not reported because *n* < 10. Statistics reported are for weighted data

*M *mean, *SD *Standard Deviation

Thirty percent of sexual minority young adults and 14% of heterosexual young adults had severe psychological distress. Thirty-six percent of sexual minority young adults and 22% of heterosexual young adults reported clinically significant anxiety symptoms.

Inequalities due to sexual orientation remained after adjusting for relevant covariates. Sexual minority young adults had significantly lower levels of social support compared to heterosexual young adults, and this association strengthened after taking into account age, gender, ethnicity, region of residence, and socioeconomic status (*β* =  − 0.38, S.E: 0.08; see Table 2). Sexual minority young adults also reported poorer self-rated health. When adjusted for sociodemographic characteristics (Models 1 to 3), sexual minority young adults had over four times the odds of reporting poor self-rated health when compared to heterosexual young adults (OR 4.78, 95% CI 2.94–7.76 in Model 3, see Table 3). This association reduced slightly after controlling for social support in Model 4, but remained strong (OR 3.91, 95% CI 2.41–6.34).

**Table 2:** Association between sexual orientation and social support

	Model 1 B (SE)	Model 2 B (SE)	Model 3 B (SE)
Heterosexual	Reference	Reference	Reference
Sexual minority	– 0.35 (0.08)***	– 0.36 (0.08)***	– 0.38 (0.08)***

Model 1 is unadjusted, Model 2 adjusts for sex and age, Model 3 additionally adjusts for ethnicity, region of residence, and equivalised household income

*** *p* < 0.001

**Table 3:** Association between sexual orientation and fair or poor self-rated health

	Model 1 O.R. (95% CI)	Model 2 O.R. (95% CI)	Model 3 O.R. (95% CI)	Model 4 O.R. (95% CI)
Heterosexual	Reference	Reference	Reference	Reference
Sexual minority	4.11 (2.47–6.84)***	4.17 (2.47–7.02)***	4.78 (2.94–7.76)***	3.91 (2.41–6.34)***

Model 1 is unadjusted, Model 2 adjusts for sex and age, Model 3 additionally adjusts for ethnicity, region of residence, and equivalised household income, Model 4 additionally adjusts for social support

***p < 0.001

Measures of mental health showed similar stark inequalities; sexual minority young adults had much higher scores of psychological distress, anxiety, and loneliness. These associations remained after adjusting for sociodemographic characteristics, and attenuated slightly in fully adjusted models which controlled for differences in social support, but remained substantial (see Table 4).

**Table 4:** Association between sexual orientation, mental health, and loneliness

	Model 1 B (SE)	Model 2 B (SE)	Model 3 B (SE)	Model 4 B (SE)
Psychological distress				
Heterosexual Sexual minority	Reference 3.18 (0.41)***	Reference 2.96 (0.41)***	Reference 2.97 (0.39)***	Reference 2.26 (0.34)***
Anxiety				
Heterosexual Sexual minority	Reference 0.65 (0.15)***	Reference 0.57 (0.16)***	Reference 0.57 (0.15)***	Reference 0.40 (0.15)**
Loneliness				
Heterosexual Sexual minority	Reference 1.10 (0.22)***	Reference 1.05 (0.22)***	Reference 1.08 (0.21)***	Reference 0.66 (0.18)***

Model 1 is unadjusted, Model 2 adjusts for sex and age, Model 3 additionally adjusts for ethnicity, region of residence, and equivalised household income, Model 4 additionally adjusts for social support

***p < 0.001

## Discussion

This study shows alarming inequalities in the self-rated health, mental health, and availability of social support among sexual minority youth compared to heterosexual/straight youth experienced during the height of the first COVID-19 lockdown restrictions in the UK. Using data from a nationally representative study of young adults in the UK, we find that young adults who self-identified with a sexual orientation or attraction other than heterosexual/straight had up to four times the odds of reporting poor self-rated health when compared to heterosexual young adults, as well as higher odds of reporting loneliness, anxiety, and psychological distress. Sexual minority young adults also reported less social support than their heterosexual peers. Adjusting for this attenuated but did not explain inequalities in health and mental health. Adjusting for socioeconomic adversity, ethnicity, and geographical location did not explain observed inequalities either. Inequalities in health and mental health across sexual orientation are a result of increased levels of social stress, including stigma, discrimination, and prejudice [20]. Findings from the Queerantine study showed an increase in reports of experienced discrimination and harassment because of one’s sexual orientation, gender identity or expression during the pandemic [2], which may partly explain the increased incidence of poor mental health reported among the sexual minority community during this period.

The present study builds on existing work that has examined sexuality-based inequalities in the UK using the same dataset. Earlier work by Amos and colleagues [21] found that large inequalities were present among sexual minority children (aged 14 years old) in terms of depressive symptoms, self-harm, life satisfaction and bullying; with similar large inequalities in terms of physical activity, self-perceived weight issues, and engagement with smoking, alcohol and cannabis, all suggesting that sexual minority children were at greater risk of harmful outcomes. Similarly, descriptive data compiled by Patalay and Fitzsimons [22] showed that sexuality-based inequalities in mental health persisted when children were aged 17, with a 2.2 point difference in average K6 scores between sexual minority young people (8.36) and heterosexual young people (6.94). Looking at the sample of young people as a whole they found that there was little change in distress scores between ages 17 and 19, although this overall trend masked pronounced rises in distress among women and a small decrease among men (no breakdown by sexuality was given) [22]. Direct comparisons with the data in the current study are challenging because of confounding between changes due to age and life course stage and changes due to the COVID-19 pandemic, as well as differences in the sample composition. In particular, we note that Patalay and Fitzsimons [22] psychological distress score is higher at age 19 (mean K6 score = 7.98) than that reported in our analytic sample (mean K6 score = 7.20). We attribute this to a different in the construction of the analytic sample, which required participants to be present in the MCS6, MCS7 and the first wave of the COVID-19 study, which led to a smaller and possibly more select sample (*N* = 1,832 vs *N* = 2,289). It is, therefore, likely that our analyses represent a slight underestimation of sexuality-based inequalities in mental health facing the MCS cohort. Furthermore, our study uses data from the first COVID-19 wave of the MCS, collected in May 2020, in the early stages of the pandemic in the UK. It is likely that with accumulated exposure to unsupportive, non-affirming environments the detrimental associations reported here have exacerbated.

Other studies conducted at later stages of the pandemic have reported findings similar to the present study that shine light on the unequal impact of the COVID-19 pandemic on sexual minority young adults, albeit with the absence of representative data [8–10]. These associations between sexuality and mental health during the pandemic are compounded by already-existent inequalities in mental health and wellbeing among sexual minority adolescents and young people, including higher levels of self-harm, suicidality, depression, and substance misuse compared to heterosexual youth [23]. The exacerbation of mental health inequalities are a cause of concern given that the lockdown entailed a separation from social networks and formal support that could help people in managing mental health issues. We had hypothesised social support to be highly protective of mental health, and despite differences emerging by sexuality, social support did little to attenuate the association between being a sexual minority and poorer mental health. We, therefore, turn to alternative factors, including experiences of discrimination, sheltering in unsupportive households and/or communities, interruptions in life course milestones of significance such as coming out and interruptions to identity formation and role exploration, concealment of identity and expression, and loss of support from sexual minority networks and organisations as potential explanatory factors between being a sexual minority and poorer mental health during the pandemic. Such factors, which are likely to be highly relevant in explaining the inequalities in physical and mental health, are rarely collected in population-based surveys such as the data used in this study.

### Strengths and limitations

In addition to the restricted sample size of the first COVID-19 wave of the MCS, other (related) limitations include that we were unable to disaggregate our estimates across different sexual minorities (for example examining bisexual young adults), or directly explore how other intersectional factors amplified or reduced the associations between sexuality and mental health. Due to limited sample sizes, we were only able to focus on sexuality, rather than exploring inequalities across the LGBTQ + community. While MCS does collect data on gender identity, which could facilitate exploration of transgender and gender diverse people (TGGD) using the main cohort study, the small numbers of respondents in this first COVID-19 wave meant that further exploration was not possible without creating a grouping that lumped sexual minority and TGGD respondents together. Given COVID-19-related studies documenting inequalities within the LGBTQ + spectrum [2], this decision was deemed to be inappropriate without the data to facilitate more granular analysis. Another limitation was that we were unable to explore the impact of sexual minority specific experiences—such as instances of homophobia or discrimination—in driving mental health inequalities. Neither were we able to build a more nuanced understanding of how different forms social support may moderate the observed associations and how these support needs may vary between sexual minorities and heterosexual people.

Our study design did not allow us to examine changes in inequalities pre- and post-pandemic. Here, we focused on establishing the magnitude of inequalities at the height of the first lockdown in the UK. The documented presence of these inequalities is enough to prompt action, irrespective of whether these have simply persisted from pre-pandemic times. In addition, it has been argued elsewhere that the presence of pre-existing inequalities has sometimes been used to explain away the importance of inequalities observed during the pandemic [24]. Our analytical framework is a deliberate choice in response to these concerns; while future researchers may explore if and how inequalities between sexual minorities attenuated or magnified during the pandemic, our intention here was to provide evidence on inequalities during COVID-19 irrespective of their temporal origin. In this respect, we feel that this evidence is more aligned with a social policy agenda that seeks to ‘build back fairer’ [25], rather than one that seeks to replicate injustices.

### Conclusions

The implications of these findings are extensive and demand prompt action. Wider socio-political othering and exclusion of “non-normative” groups, including sexual minority youth, is a distal determinant of the inequalities reported here. Processes of exclusion have been tangible in the management of the pandemic by the UK government, who have disregarded the vulnerability of sexual minority young people to the negative consequences of lockdown and social distancing regulations. In their efforts to “build back fairer,” a concerted effort to inclusive policy should recognise the role of families, communities, and institutions in challenging heteronormative and cis-normative discourses, and supporting sexual minority young people. Examples include a stronger provision of safe spaces in the community and in institutions, policies that address marginalisation and harassment (including online) of sexual minority populations, and increased support for charities and organisations that work with and for the community.

## Data Availability

MCS is deposited with the UK Data Service at the University of Essex.

## Code availability

Analysis code can be made available on request.
